# Current perspectives in the treatment of resistant schizophrenia

**DOI:** 10.4103/0019-5545.58289

**Published:** 2009

**Authors:** R. K. Solanki, Paramjeet Singh, Deepti Munshi

**Affiliations:** Department of Psychiatry, Psychiatry Center, SMS Medical College, Jaipur, India

**Keywords:** Adjuvant therapy, clozapine, treatment resistance

## Abstract

This article summarizes the current knowledge base on the diagnosis and management of treatment resistant schizophrenia. While the prevalence of treatment resistant schizophrenia is definition dependent, estimates have ranged from 30% to up to 60%. This article first looks into the various diagnostic criteria of treatment resistant schizophrenia. Then the literature is reviewed about the pharmacotherapeutics of its management. Clozapine emerges to be the gold standard. In addition risperidone and high dose olanzapine also emerge as clinically useful options. Other emerging adjunctive treatment options are equally addressed.

## INTRODUCTION

Knowledge base regarding medical treatment of schizophrenia is rapidly expanding. Information about agents with established antipsychotic properties is increasing, more and more agents with putative antipsychotic properties are being identified yet the response to remission strategies are still to be developed so far and the residual symptoms have become cause of concern in incomplete responders or resistant cases.

It was clear that a specific group of patients remained symptomatic in spite of treatment, and therefore were considered refractory or resistant to phenothiazines.[1]

Even now it is assumed that 20-30% of patients who have schizophrenia do not respond to treatment with conventional antipsychotics, but some authors have mentioned higher rates (up to 60%).[2–5]

Although clozapine use has often been restricted to treatment resistant cases, the benefit it bestows outweighs the potential risk of side-effects in patients with less stringently defined treatment resistance. The concept of Treatment Resistant Schizophrenia is more important today than ever before because:

1. It provides a guide to treatment choice in an era where the options to treat schizophrenia, at least from a pharmacological perspective, have never been more abundant.
2. It provides the impetus to develop more effective treatments for those aspects of schizophrenia which current treatments fail to address adequately for some or all patients, for example, negative symptoms and cognitive functions.
3. Finally, treatment-resistant schizophrenia can be the basis for identification of a subgroup of schizophrenia patients who have a unique etiology, possibly on a genetic basis or that of some gene environment interaction. This can not only lead to the development of effective treatments for these individuals but also, by reducing heterogeneity, enhance the ability to study the etiology of schizophrenia in those who do respond adequately to current treatments.[5]

Prior to assigning a patient with the label of treatment resistance, the concept and definition of treatment resistant schizophrenia should be well defined. Even though many authors have tried to construct operational definitions (unidimensional approach) on a single criteria e.g. Csernansky and collegues,[6] bi-dimensional scale by May *et al.,*[7] Brenner *et al.,*[8] Brenner and Merlo.[9] The operational criteria most widely used for the definition of TRS in clinical studies are those of Kane and colleagues[3] which enabled the selection of patients who had TRS for the study that introduced clozapine to the therapeutic armamentarium for schizophrenia, thereby paving the way for the emergence of second-generation antipsychotic agents (SGAS). Kane's criteria are three dimensional, meaning that to be considered refractory; the patient must fulfill the following criteria or dimensions:

### Historical

At least three treatments with antipsychotics of at least two different chemical classes with doses equivalent to 1000 mg/d of chlorpromazine for a period of 6 weeks, without significant relief.

No period of good function within the preceding 5 years.

### Actual

A score of at least 45 in the BPRS (1-7 degrees of severity) with scores of at least 4 in two of the following items: Conceptual disorganization, suspiciousness, hallucinatory behavior, or unusual thought content

CGI >or = 4 (moderately ill)

### Prospective

No improvement after 6 weeks of treatment with haloperidol (up to 60 mg/d or higher); improvement is defined as A 20% reduction of the BPRS as compared with the level of severity defined by the actual criteria and/or A post treatment CGI of ≤ 3 or a BPRS ≤ 35.

The criteria for neuroleptic resistance utilized in the U.S. multicenter clozapine trial have been criticized as too narrow, imprecise and vague,[10–13] specially with respect to the duration of treatment, adequate explanation of the disabling effects of cognitive dysfunction and negative symptoms and the issue of adequate dosing.

An international study group to define treatment resistance in schizophrenia proposed a multidimensional approach, including psychopathology (positive and negative symptoms), functional disability, and behavior. They incorporated the following dimensions and provided a useful rating scale to measure treatment responsiveness on a continuum.[12]

1. Persistent moderate, severe positive, negative disorganization symptoms.
2. Cognitive dysfunction in multiple spheres which interfere with work and social function.
3. Recurrent mood disturbance and suicidality.
4. Poor work and social function.
5. Poor (subjective) quality of life.
6. Bizarre behavior.
7. One adequate trial of a typical neuroleptic which is tolerable to the patient at doses of 2-20 mg/day of haloperidol or equivalent for duration of 6-12 weeks. To simplify clinical decision making, a number of treatment guidelines, such as the American Psychiatric Association[14] guidelines and the Schizophrenia Patient Outcomes Research Team[15] guidelines, or algorithms, such as the Texas Medication Algorithm Project,[16] state that a patient who has not responded to two or three treatments using atypical antipsychotics for a duration at least 4 to 6 weeks can be considered as having TRS and is eligible for treatment with clozapine. The most recent algorithm, the Schizophrenia Algorithm of the International Pharmacological Algorithm Project (www.ipap.org) states that a patient who has not responded to two trials of 4-6 weeks' duration using monotherapy with two different SGAs (or two trials with an FGA, if SGAs are not available is considered to have TRS and is eligible for treatment with clozapine, for a six month trial with doses up to 900 mg/d.

## TREATMENT OF RESISTANT SCHIZOPHRENIA WITH ATYPICAL ANTIPSYCHOTIC DRUGS

### Clozapine

The most consistent results regarding efficacy in this group of patients have been observed with clozapine. The data from open and controlled studies show superior effects of clozapine on positive and negative symptoms, compared to prior treatment with typical neuroleptics.[13,17–20] It has been found that up to 60% of neuroleptic resistant patients treated with clozapine respond to it, half of these respond within 6 weeks of treatment, while the other half respond within 6months of treatment.

Meta-analyses of controlled trials involving patients who had TRS[21–24] and a systematic review[25] showed that clozapine is as effective or is more effective than other SGAs for treating TRS. Wahl beck and colleagues'[21] metaanalysis included 30 trials of clozapine versus an FGA. Chakos and colleagues[22] meta-analysis reviewed trials of SGAs versus FGAs and also reviewed trials of SGAs versus other SGAs. Taylor and Duncan-McConnell[25] systematically reviewed controlled trials of an SGA versus an FGA.

One of these studies,[23] which compared clozapine with an FGA, challenged these results, criticizing methodological bias (e.g., the heterogeneity and duration of the studies, the initial psychopathology of patients, the year of publication, and sponsorship) in the clinical trials but still found a 0.44 effect in favor of clozapine.

The meta-analysis of the Cochrane Center included only eight studies comparing clozapine with an SGA. This meta-analysis found a trend for clozapine to be more effective in improving positive symptoms but not negative symptoms; no differences were seen in other outcome variables, such as relapse rates or global improvement. There was a trend for an SGA to be more effective in improving cognition.

Two famous meta-analyses showed opposite results in terms of the efficacy of SGAs over FGAs. In one of these studies Geddes and colleagues[26] found that the superiority of an FGA is related to the dosage of the comparator (i.e., when the dose of haloperidol was 12 mg or more, the SGA had no superiority over the FGA in efficacy or tolerability). Some of the studies in this meta-analysis involved controlled trials in which patients who had TRS were treated with clozapine, but no specific conclusions about clozapine treatment were reported.

Davis and colleagues[27] challenged these results with another metaanalysis showing that clozapine had almost twice the effect size (0.49) of some other SGAs (0.29 for amisulpride; 0.25 for risperidone; 0.21 for olanzapine).

Solanki and colleagues in a review concluded that clozapine exhibits superiority over typical antipsychotics in terms of both efficacy (as shown by an improvement in overall psychopathology) and safety. However the magnitude of its effect is not consistent. Efficacy data for other SGA's in the treatment of refractory schizophrenics were inconclusive.[28]

Two recent important pragmatic trials support the effectiveness of clozapine over SGAs for the treatment of schizophrenia. Evoy and colleagues,[29] in a phase II trial in the Clinical Antipsychotic Trials of Intervention Effectiveness (CATIE) study[30] (which involved about 1400 patients), studied 99 patients who had not responded to atypical antipsychotics in previous phases of the trial because of lack of efficacy.[31] Patients were assigned randomly to open-label clozapine (n = 49) or to blinded treatment with another SGA (olanzapine, n = 19; quetiapine n = 15; or risperidone n = 16). When compared with other SGAs, clozapine had the greatest reductions in the PANSS total score and the lowest discontinuation rates; that is, the use of clozapine proved to be more effective than switching to another SGA in patients who previously had not responded to another SGA.

In another pragmatic trial Lewis and colleagues[32] studied 136 patients who had schizophrenia and who had shown a poor response to two or more antipsychotic medications. The patients were assigned randomly to receive clozapine (n = 67) or an SGA (risperidone, olanzapine, quetiapine, amisulpride) (n = 69). At the end of 1 year patients who received clozapine showed a reduction in psychopathology (PANSS score) and improved quality of life. The authors state that patients who have schizophrenia and who have had a previous poor response to two or more antipsychotic drugs should receive clozapine instead of another SGA.

### Clozapine and ECT

There is some evidence that ECT can augment the response to clozapine. A review[33] of 36 published cases of clozapine treatment combined with ECT reports that 67% of patients benefited from the combination. The indications for combination treatment in these cases were resistance or intolerance to typical neuroleptics or resistance to clozapine or ECT alone. The number of ECT sessions was 12 ± 6, clozapine dose during ECT was 385 ± 172 mg/day. The procedure was reported to be safe and well tolerated. Kupchik *et al.*[33] suggested that the efficacy of the combination treatment was based on mechanisms such as clozapine induced lowering of the seizure threshold or ECT compromising the blood brain barrier so that greater amounts of clozapine penetrate into the brain. Maintenance ECT strategies with combination treatment, especially studies with duration of follow up ≥6 months, are needed.

### Clozapine augmentation

A narrow definition of partial or incomplete response to clozapine is the persistence of psychotic symptoms despite a trial of clozapine with adequate doses (i.e., 300-900 mg/d) for a minimum of 8 weeks and for up to 6 months with plasma levels reaching 350 ng/ml.[34] Thus the improvement of psychotic symptoms is considered the main treatment target, and, as an apparent logical consequence, the addition of high-potency antipsychotic medications to clozapine has been proposed for the treatment of these symptoms.

Various antipsychotic agents have been used, supposedly to augment the antipsychotic properties of clozapine: amisulpride,[35–40] aripiprazole,[41–44] haloperidol,[45] loxapine,[46] olanzapine,[47] pimozide[48] and ziprasidone.[49] The benefit of these augmentation strategies remains inconclusive because they were tested in case series or case reports, which have a low strength of evidence as compared with controlled trials.[50,51] More robust evidence is derived from four placebo-controlled trials, one with sulpiride[52] and three with risperidone.[53–56] The study by Shiloh and colleagues[52] showed a significant improvement in positive and negative symptoms in the group that received clozapine plus sulpiride when compared with placebo group. It was suggested that this effect could be explained by the selective enhancement of D2 blockage by sulpiride. It is well known that risperidone has a strong affinity for D2 receptors, but the hypothesis that the blocking these receptors would improve persistent positive symptoms in patients resistant to clozapine was supported only by the study by Josiassen and colleagues.[53] The studies by Yagcioglu[54] and by Honer[55] found no differences between the risperidone and placebo groups. Therefore these studies did not support the hypothesis that adding a more potent antipsychotic to enhance or optimize D2 affinity would improve psychotic symptoms in persons who respond poorly to clozapine. In the study by Yagcioglu and colleagues,[54] the placebo group showed a greater reduction in PANSS positive scores than the risperidone group, and the neuropsychological evaluation of the same sample published in a subsequent study showed no benefit of risperidone in terms of cognitive functions.[57]

There is also some evidence to support the addition of omega-3 triglycerides (2-3g EPA) in non or partial responders to antipsychotics including clozapine.[58,59]

Topiramate has also been suggested, either to augment clozapine or to induce weight loss. It may be effective as augmentation[60] but can worsen psychosis.[61,62]

Some studies have shown that Lamotrigine in doses of (25-300 mg/day)[61–64] may be useful in partial or non responders. It has also shown to reduce alcohol consumption in some.[65] Previous experimental data suggests that excessive glutamate neurotransmission contributes to the positive symptoms of schizophrenia. In a randomized placebo-controlled crossover trial by Tiihonen *et al.,* 200 mg/d of lamotrigine was gradually added to the ongoing clozapine treatment of 34 TRS patients. Clinical assessments were made by the Positive and Negative Syndrome Scale at the beginning and end of each treatment period. In intention-to-treat analysis, lamotrigine treatment was more effective in reducing positive (effect size.7, *P* = .009) and general psychopathological (effect size.6, *P* = .030) symptoms, whereas no improvement was observed in negative symptoms.[64]

Finally, when clozapine augmentation with antipsychotics fails, switching to another antipsychotic has been proposed. This strategy is considered to have a weak level of evidence.[4]

### Olanzapine

Olanzapine was the antipsychotic most tested in four open trials.[66–69] The results of controlled trials of olanzapine efficacy for treatment resistant patients are inconsistent. Breier and Hamilton[70] reported that a significantly greater proportion of treatment resistant patients responded to olanzapine (47.4%) than haloperidol (34.9%). They noted greater improvement in positive, negative and depressive symptoms in olanzapine treated group compared to the control group. However Coloney *et al.,*[71] found no difference in efficacy between the patients treated with olanzapine or chlorpromazine group. The response rate in both groups was quite low. This study included patients who had been previously treated with risperidone and clozapine as well as typical neuroleptics. Open label studies of olanzapine by Martin *et al.*[72] and Dursun *et al.,*[73] have shown that olanzapine at moderate to high doses 25-40 mg/day is effective for a significant proportion of treatment resistant patients, However a study by Sanders and Mossman[74] found that olanzapine is not effective in chronic, severe treatment resistant patients (doses 10-20 mg/day). In a randomized double blind comparison study of clozapine and high dose olanzapine (25-45 mg/day) by Meltzer *et al.,*, olanzapine at higher than customary doses demonstrated similar efficacy to clozapine in treatment-resistant schizophrenia and schizoaffective disorder. There was significant improvement in multiple measures of psychopathology, mainly between 6 weeks and 6 months of treatment was found in both treatment groups, with no significant difference between the 2 treatments except for the Global Assessment of Functioning score, which favored clozapine. Improvement in some domains of cognition was significant and equivalent for both drugs as well.[75]

### Risperidone

Early open and non blinded studies on treatment resistant schizophrenic patients with risperidone by Smith *et al.,*[76] indicated its effectiveness on positive but not negative symptoms. A double blind study by Wirshing *et al.,*[77] reported significantly higher improvement in BPRS total score during a fixed dose (6 mg/day) phase comparing risperidone with haloperidol. The predictors of robust response were more severe positive symptoms, conceptual disorganization, less depression, akathisia and tardive dyskinesia.

### Risperidone v/s clozapine

Bondolfi *et al.,*[78] double blind study found clozapine and risperidone equally effective to reduce psychotic symptoms (risperidone 6 mg/d and clozapine 300 mg/d for 1 week then adjusted). At the end point 67% of the risperidone group and 65% of the clozapine group responded (20% reduction in PANSS score). This study has been criticized on multiple grounds and does not constitute evidence for equivalence of these drugs. An open non blinded prospective study by Flynn *et al.,*[79] found clozapine to be superior to risperidone as determined by CGI, PANSS total score, positive subscale and factors of psychomotor retardation, psychosocial withdrawal and excitement. The open nature of the study and small sample size 57 in clozapine group and 29 in risperidone group might have affected the outcome. Another non blinded study conducted by Lindenmayer *et al.,*[80] found that clozapine showed superior efficacy on all outcome measures but the difference did not reach statistical significance, suggesting that the study was underpowered.

In conclusion although there is some evidence that risperidone is effective in some treatment resistant cases, its efficacy equal to that of clozapine has not been shown.

### Quetiapine

Only a few case reports suggest that some treatment resistant patients respond to quetiapine.[81,82]

### Aripiprazole

Moderate effect of Aripiprazole (15-30 mg/day) has been seen in patients resistant to risperidone or olanzapine.[83] Higher doses have also have been used.[84] Chang *et al.,*[85] studied augmentation of clozapine with aripiprazole and found significant improvement in the negative symptoms, and significantly lower level of prolactin and triglycerides in the patients treated with the combination.

### Ziprasidone

In a 52 week, open label continuation study of Ziprasidone in Treatment Resistant Schizophrenia, ziprasidone at a modal dose of 160mg/day, was effective in maintaining symptom control over a one year continuation study period.[85]

## ADJUNCTIVE TREATMENT

1. Lithium: Studies by Shiloh *et al.,*[87] that lithium as an adjunct may enhance the efficacy of antipsychotic medication. Additionally it may be effective for affective symptoms, impulsivity, or violent behavior.
2. Anticonvulsants: Manic, impulsive or violent behavior may respond well to the addition of carbamezapine or valproic acid.[88–90]
3. Antidepressants: Residual negative symptoms, obsessive compulsive symptoms and other anxiety symptoms besides depression, respond to SSRIs.[91]
4. Beta blockers: High dose propranolol up to 1200 mg/day has been shown to augment antipsychotic efficacy in treatment refractory schizophrenia. It may produce its beneficial effect by its ability to treat akathisia, increasing antipsychotic serum levels, decreasing anxiety symptoms.[92]
5. Glycine and D cycloserine: Much interest has surrounded the use of glycine and partial agonists acting through the glycine site on NMDA receptors in the treatment of negative symptoms in schizophrenia. High doses of glycine (30-60 mg/day) have shown improvement in negative symptoms when added to antipsychotic medication.[93,94]
6. Galantamine: Because of the demonstration of a selective alpha nicotinic receptor abnormality in patients with schizophrenia, galantamine was added to the stable regimen of atypical and other antipsychotic medication in a study on a single patient by Rosse *et al.* Initially in doses of 4 mg bid (1^st^ week) which was increased up to 12 mg bid. Galantamine is distinguished from other acetyl cholinesterase inhibitors by its positive allosteric modulatory properties, improving the efficiency of transduction of the acetylcholine signal at nicotinic receptors. This latter property may have contributed to the observed improvement in negative symptoms observed in this study Importantly, positive symptoms were unchanged during the 2-month trial.[95]

## Role of poly-pharmacy

Although poly-pharmacy is widely practiced in the clinical setting, it is not recommended in the existing practice guidelines. As described earlier various studies have described efficacy of different combinations but no study has compared relative benefits of different combinations. In a meta-analysis Correll *et al.,*[96] found that antipsychotic medication co-treatment was superior to monotherapy.

Despite encouraging results role of combinations of medication require further exploration.

## Psychosocial interventions

Role of psychosocial interventions i.e. psychotherapy, family therapy, CBT and occupational therapy (OT) in management of schizophrenia is well established. But in treatment resistant case these interventions are less studied. In a review Hellewell[97] found that combination of clozapine with a psychosocial intervention widely modifies the outcome. Studies[98–100] have shown that Cognitive-behavioral therapy is useful in treating this group of the patients. In a recent review Rathod and colleagues[101] found CBT as an effective adjuvant to antipsychotic medication in the treatment of medication resistant schizophrenia. Another meta-analysis[102] has underlined the importance of family intervention and cognitive behavior therapy. In a comparative study Randal *et al.,*[103] studied improvement in symptom and function with recovery-focused multimodal psychotherapy in treatment-resistant psychotic illness and found significant improvement. In a Randomized controlled trial Buchain *et al.,*[104] investigated effectiveness occupational therapy in patients with treatment-resistant schizophrenia, they found the combination of OT and clozapine to be more effective than the use of clozapine alone.

## CONCLUSION

Amidst the diagnostic dichotomy of the various criteria explaining treatment resistance, substantial positive symptom clusters have been addressed in most studies, however negative symptoms and cognitive impairment and their influence on the Quality Of Life and executive functioning of the patient are undermined.

Though clozapine appears to emerge as the gold standard for treatment resistant schizophrenia, however it still lacks effect in some of the subjects. Nevertheless augmentation studies have shown promising results. Other atypical antipsychotics esp. Risperidone and high dose Olanzapine also show response in some resistant cases.

Studies till date indicate that psychosocial interventions have important role in the management of the patients of treatment resistant schizophrenia and this field needs more exploration.

The reason for this treatment resistance is not clearly addressed but the role of rapid metabolisers, pharmacogenetics and single nucleotide polymorphisms may explain the same in the time to come. Remission is a realistic goal for patients with Schizophrenia and if sustained remission without relapse can be achieved then patients may attain functional recovery.
